# Supplementary material for: Global glacier-free topography reveals a large potential for future lakes in presently ice-covered terrain
Source: Nat Commun. 2026 May 2;17:3985. doi: 10.1038/s41467-026-72548-9 (PMC13135513; doi:10.1038/s41467-026-72548-9)
Supplement: Supplementary file 1 — Supplementary information [file 41467_2026_72548_MOESM1_ESM.pdf]

Supplement to:

Global glacier-free topography reveals large potential for future lakes in presently ice-covered terrain

T. Frank[1,*], W. van Pelt[1], D.R. Rounce[2], G. Jouvet[3], and R. Hock[4,5]

[1]Department of Earth Sciences, Uppsala University, Uppsala, Sweden

[*]Corresponding author: thomas.frank@geo.uu.se

[2]Department of Civil and Environmental Engineering, Carnegie Mellon University, Pittsburgh, PA, USA

[3]Institute of Earth Surface Dynamics, University of Lausanne, Lausanne, Switzerland

[4]Department of Geosciences, University of Oslo, Oslo, Norway

[5]Geophysical Institute, University of Alaska Fairbanks, Fairbanks, AK, USA

Table S1: Global and regional glacier volumes, SLE and mean area-weighted thickness h_{mean} alongside glacier-covered area [from RGI v6.0; 1, 2] and number of glaciers (n) compared to two recent studies. Percentages in parentheses refer to difference between this study's volumes and those from the other studies. Note that the volumes by [3] refer to the homogenized and corrected numbers calculated by [4]. Volume and SLE numbers of this study refer roughly to the year 2013.

Region	Area (km^2)	n	SLE (mm)	h$_{mean}$ (m)	Volume ($\times 10^3$km^3)		
					This study	Farinotti et al. (2019)	Millan et al. (2022)
01-Alaska	86725	26813	46.9 ± 21.0	238 ± 106	20.63 ± 9.22	20.03 ± 5.19 (+8.2%)[1]	19.16 ± 5.60 (+13.2%)[1]
02-W Canada & US	14524	17991	2.6 ± 0.5	72 ± 14	1.05 ± 0.21		
03-Arctic Canada N	105111	4527	51.4 ± 8.2	212 ± 34	22.29 ± 3.58	28.33 ± 7.35 (-21.3%)	25.40 ± 7.23 (-12.2%)
04-Arctic Canada S	40888	7390	16.9 ± 1.5	169 ± 15	6.92 ± 0.62	8.61 ± 2.23 (-19.6%)	7.03 ± 2.11 (-1.6%)
05-Greenland Periphery	89717	19233	28.8 ± 2.2	139 ± 10	12.46 ± 0.94	15.69 ± 4.07 (-20.6%)	12.54 ± 3.95 (-0.6%)
06-Iceland	11060	568	8.8 ± 1.4	325 ± 52	3.60 ± 0.57	3.77 ± 0.98 (-4.5%)	3.73 ± 0.90 (-3.5%)
07-Svalbard	33959	1615	15.1 ± 1.3	202 ± 17	6.85 ± 0.58	7.47 ± 1.94 (-8.3%)	6.86 ± 2.26 (-0.1%)
08-Scandinavia	2949	3391	0.8 ± 0.1	109 ± 14	0.32 ± 0.04	0.30 ± 0.08 (+6.7%)	0.32 ± 0.11 (0.0%)
09-Russian Arctic	51592	1068	23.8 ± 4.0	213 ± 36	10.97 ± 1.84	14.64 ± 3.80 (-25.1%)	15.57 ± 3.92 (-29.5%)
10-North Asia	2410	4947	0.3 ± 0.1	50 ± 12	0.12 ± 0.03	0.14 ± 0.04 (-14.3%)	0.14 ± 0.06 (-14.3%)
11-Central Europe	2092	3678	0.3 ± 0.0	48 ± 5	0.10 ± 0.01	0.13 ± 0.03 (-23.1%)	0.12 ± 0.05 (-16.7%)
12-Caucasus & Middle East	1307	1510	0.2 ± 0.1	54 ± 23	0.07 ± 0.03	0.06 ± 0.02 (+16.7%)	0.07 ± 0.04 (0.0%)
13-Central Asia	49303	53924	6.9 ± 1.1	57 ± 9	2.81 ± 0.43	7.01 ± 1.82 (-8.8%)[2]	9.48 ± 3.74 (-32.6%)[2]
14-South Asia W	33568	27637	5.9 ± 1.8	71 ± 22	2.38 ± 0.73		
15-South Asia E	14734	13024	2.9 ± 1.2	81 ± 33	1.20 ± 0.48		
16-Low Latitudes	2341	2830	0.2 ± 0.1	34 ± 26	0.08 ± 0.06	0.10 ± 0.03 (-20.0%)	0.08 ± 0.05 (0.0%)
17-Southern Andes	29429	15572	16.6 ± 3.8	235 ± 53	6.92 ± 1.56	5.34 ± 1.39 (+29.6%)	6.03 ± 1.64 (+14.8%)
18-New Zealand	1162	3363	0.1 ± 0.0	43 ± 9	0.05 ± 0.01	0.07 ± 0.02 (-28.6%)	0.07 ± 0.03 (-28.6%)
19-Subantarctic & Antarctic Islands	132867	2061	79.1 ± 13.0	381 ± 63	50.57 ± 8.34	46.47 ± 12.06 (+8.8%)	35.10 ± 9.10 (+44.1%)
**Total**	705738	211142	307.7 ± 60.2	212 ± 41	149.41 ± 29.28	158.16 ± 41.04 (-5.5%)	141.70 ± 40.77 (+5.4%)

[1] Sum of regions 1 and 2 as given in [4].
[2] Sum of regions 13, 14 and 15 as given in [4].

Table S2: Contributions to the total error σ_V by the individual terms given in eqs. (5), (6), (S2). σ_V denotes the total volume error in absolute numbers, $\frac{\sigma_V}{V}$ is the same but relative to the regional volume, $\frac{\sigma_A}{A}$ is the error in the outlines relative to regional glacier area, $\frac{\sigma_h}{h}$ is the error in mean ice thickness relative to regional mean thickness, σ_h is the same but in absolute numbers, σ_C and μ_C together reflect an error from calibration, σ_{obs} is a systematic absolute error in thickness observations and σ_{repr} is the absolute error from biases in the sample of observed glaciers.

Region	σ_V ($\times 10^3$ km^3)	$\frac{\sigma_V}{V}$	$\frac{\sigma_A}{A}$	$\frac{\sigma_h}{h}$	σ_h (m)	σ_C (m)	μ_C (m)	σ_{obs} (m)	σ_{repr} (m)
01-Alaska	9.22	0.45	0.05	0.44	105.65	71.36	-100.00[1]	2.00	31.69
02-W Canada & US	0.21	0.20	0.05	0.19	13.76	21.69	9.97	2.00	8.24
03-Arctic Canada N	3.58	0.16	0.05	0.15	32.34	63.62	-30.81	2.00	9.21
04-Arctic Canada S	0.62	0.09	0.05	0.07	12.50	50.77	-6.42	2.00	10.04
05-Greenland Periphery	0.94	0.08	0.05	0.06	5.45	28.74	-3.83	2.00	3.22
06-Iceland	0.56	0.16[2]	0.05						
07-Svalbard	0.58	0.08	0.05	0.07	13.62	60.51	-7.68	2.00	10.24
08-Scandinavia	0.04	0.11[3]	0.03[3]						
09-Russian Arctic	1.84	0.17[2]	0.05						
10-North Asia	0.03	0.26	0.05	0.26	12.88	14.94	8.49	2.00	5.84
11-Central Europe	0.01	0.12	0.05	0.11	5.28	14.34	3.27	2.00	3.55
12-Caucasus & Middle East	0.03	0.41	0.05	0.41	21.79	16.07	17.29	2.00	6.53
13-Central Asia	0.43	0.15	0.05	0.14	8.23	17.10	-3.89	2.00	5.39
14-South Asia W	0.73	0.31[2]	0.05						
15-South Asia E	0.48	0.40	0.05	0.39	32.15	24.43	-12.65	2.00	16.51
16-Low Latitudes	0.06	0.69	0.05	0.69	23.50	10.25	22.85	2.00	3.36
17-Southern Andes	1.56	0.23	0.05	0.22	51.81	70.54	19.92	2.00	44.18
18-New Zealand	0.01	0.25	0.05	0.25	10.55	12.91	-1.22	2.00	4.75
19-Subantarctic & Antarctic Islands	8.34	0.16	0.05	0.16	59.79	114.18	-10.28	2.00	58.12

[1] Bounded to -100 m instead of the actual value μ_C = -200.94 m as described in the text.
[2] Regions without point observations of ice thickness in the GlaThiDa, with $\frac{\sigma_V}{V}$ being twice the uncertainty of the regions that were used to generate their model parameters.
[3] Values taken from [5]

Table S3: Calibrated model parameters as obtained through Bayesian optimization with Gaussian processes (eq. (4)) by RGI region. η is ice viscosity, c is the friction coefficient, θ is a regularization parameter, f_u is a multiplier for observed velocities and a is frontal ablation. Note that not all parameters were calibrated in all regions as described in the text.

Region	η (MPa^{-3} y^{-1})	c (MPa y$^{1/3}$ m$^{-1/3}$)	θ	f_u	a (m w.eq. yr^{-1})
01-Alaska[1]	70.00	0.050	0.30	1.00	
02-W Canada & US	35.25	0.070	0.27		
03-Arctic Canada N	70.33	0.090	0.24	0.90	
04-Arctic Canada S	63.99	0.077	0.28	0.67	
05-Greenland Periphery	41.51	0.061	0.21	0.50	
06-Iceland	58.52	0.044	0.22	0.54	
07-Svalbard	75.52	0.027	0.23	0.58	
08-Scandinavia[2]					
09-Russian Arctic	75.52	0.027	0.23	0.58	
10-North Asia	16.68	0.053	0.11		
11-Central Europe	47.75	0.058	0.27		
12-Caucasus & Middle East	37.49	0.097	0.25		
13-Central Asia	66.91	0.091	0.24		
14-South Asia W	66.91	0.091	0.24		
15-South Asia E	54.74	0.340	0.13		
16-Low Latitudes	29.26	0.055	0.11		
17-Southern Andes	49.98	0.074	0.07	0.55	
18-New Zealand	55.25	0.040	0.26		
19-Subantarctic & Antarctic Islands	25.01	0.093	0.17	0.51	0.42

[1] Parameters chosen manually as described in the text.
[2] Parameters from [5], c.f. this paper for details.

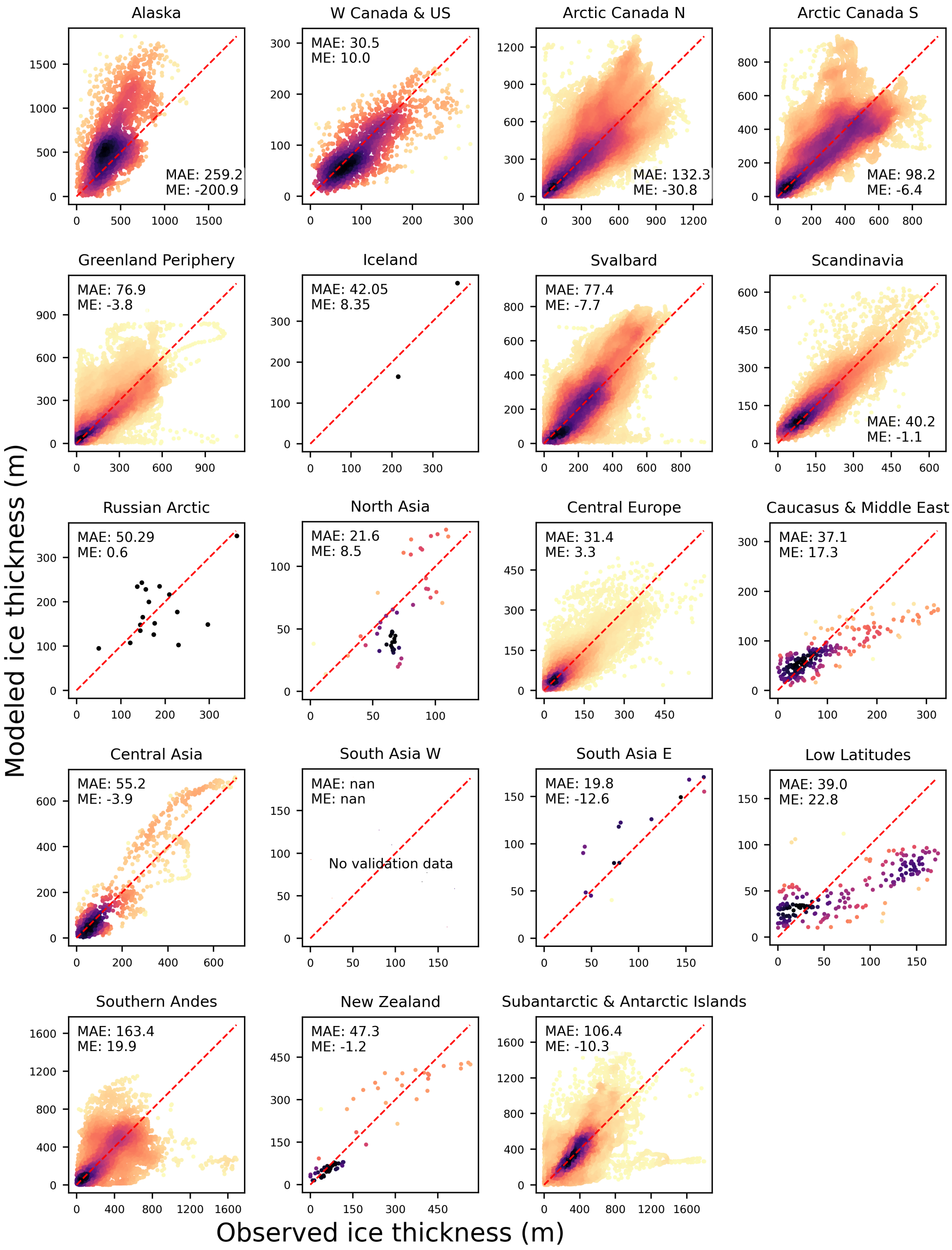

Figure S1: Modeled versus observed ice thicknesses (m) for all Randolph Glacier Inventory glacier regions with performance metrics (MAE: Mean Absolute Error, ME: Mean Error). Point color indicates point density, with darker colors showing a larger overlap of points. Red dashed line indicates 1:1 line. For Iceland and Russian Arctic, glacier mean thicknesses are shown since no point observations exist in the Glacier Thickness Database. In these two regions, performance metrics are calculated relative to mean glacier thicknesses reported in the Glacier Thickness Database.

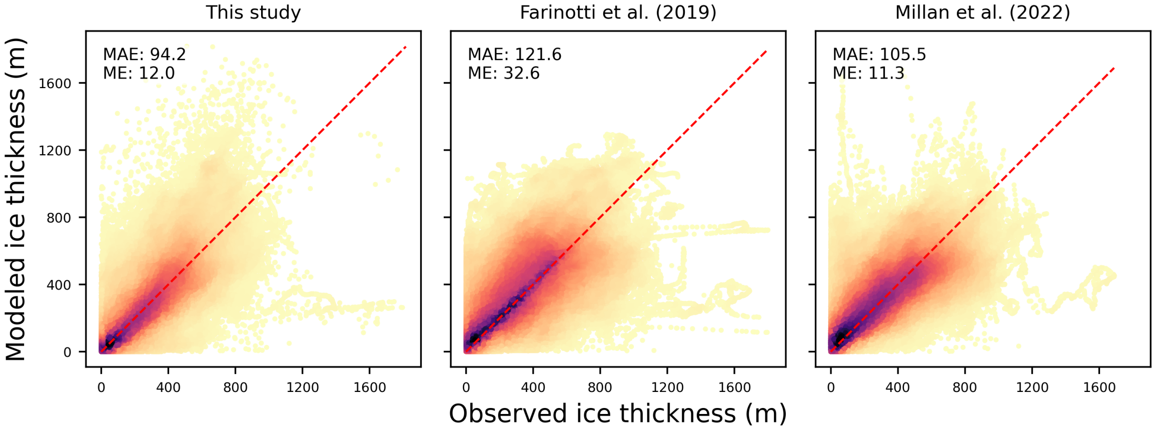

Figure S2: Modeled versus observed ice thicknesses (m) aggregated globally for this study and existing products by [6] and [3] with metrics (MAE: Mean Absolute Error, ME: Mean Error, unit: m). Point color indicates point density, with darker colors showing a larger overlap of points. Red dashed line indicates 1:1 line.

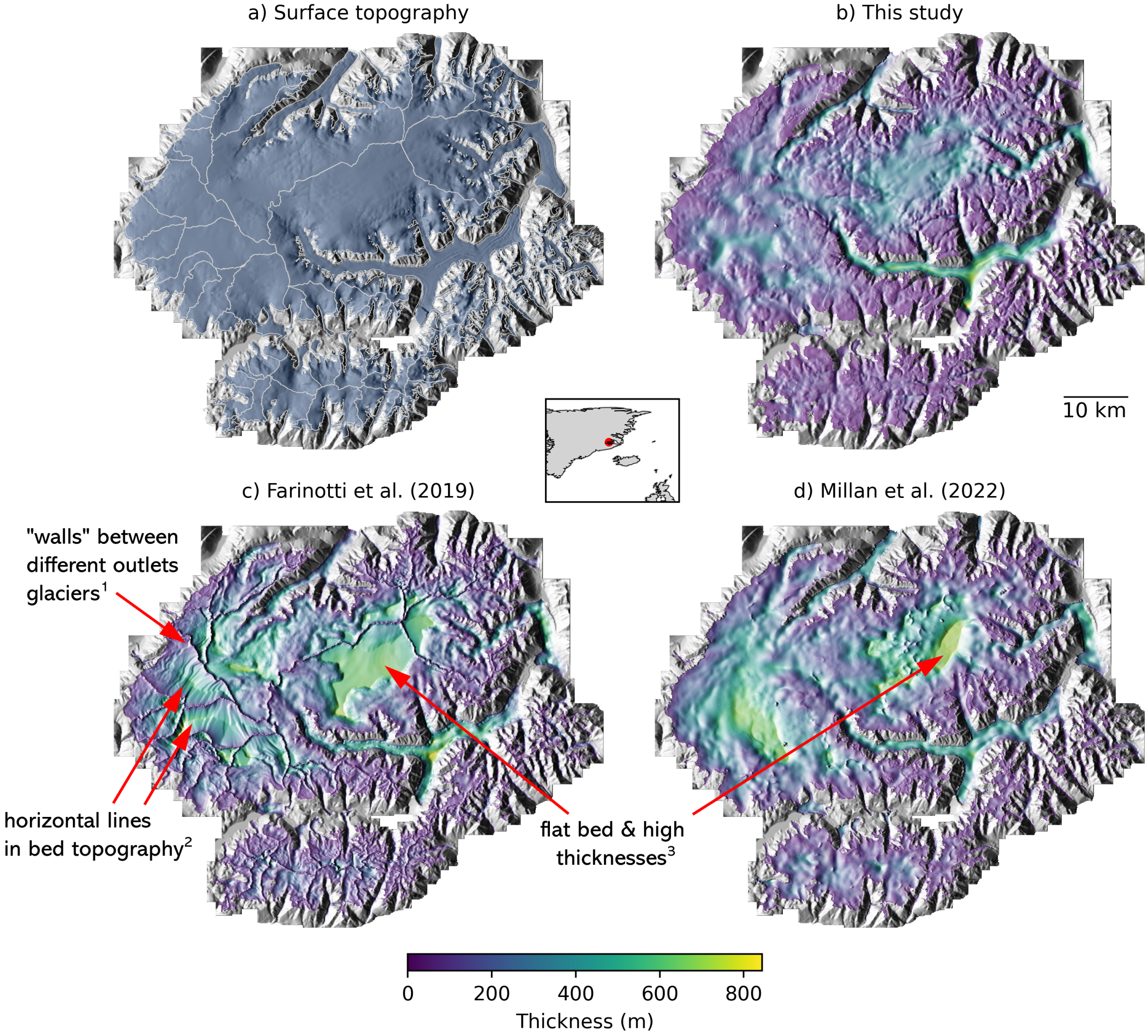

Figure S3: Comparison of simulated bed topography with existing products (central coordinates of figure: 26.78°W 71.22°N). a) Hillshade of surface topography with glacier extent in blue, and boundaries of individual outlet glaciers in light gray. b-d) Hillshade of bed topography from b) this study, c) [7] and d) [3] with modeled ice thickness draped over it. Thickness in c) and d) are calculated as the difference between observed surface elevation and modeled thickness. Inset map shows location of ice cap in Greenland. Annotations highlight examples of unrealistic bed shape: [1] is the result of modeling individual outlet glaciers separately and prescribing a zero ice thickness at glacier boundaries, seen in c); [2] is due to a flow-line approach where thicknesses away from the central flow-line are interpolated in elevation bins, seen in c); [3] is because under shallow ice flow approximations, thicknesses approach infinity as the surface slope becomes small - to avoid this, a manual correction is applied that assigns a uniform thickness in such flat areas, seen in c) and d).

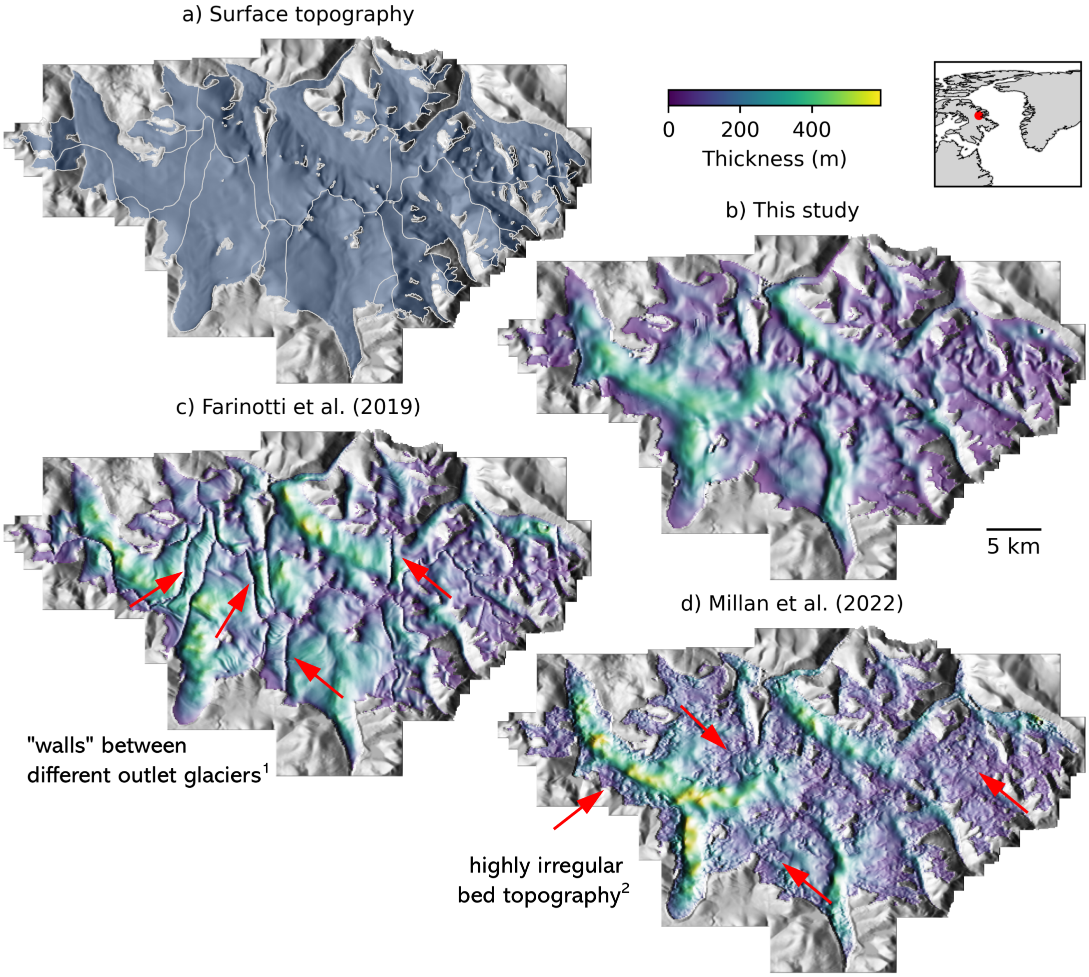

Figure S4: Comparison of simulated bed topography with existing products (central coordinates of figure: 69.70°W 69.39°N). a) Hillshade of surface topography with glacier extent in blue, and boundaries of individual outlet glaciers in light gray . b-d) Hillshade of bed topography from b) this study, c) [7] and d) [3] with modeled ice thickness draped over it. Thickness in c) and d) are calculated as the difference between observed surface elevation and modeled thickness. Inset plot shows location of glacier in Arctic Canada South. Annotations highlight examples of unrealistic bed shape: [1] is the same as in Fig. S3, seen in c); [2] is because the modeled thicknesses are directly inferred from observed velocities which can be noisy in slow-flowing areas, resulting in noisy beds seen in d).

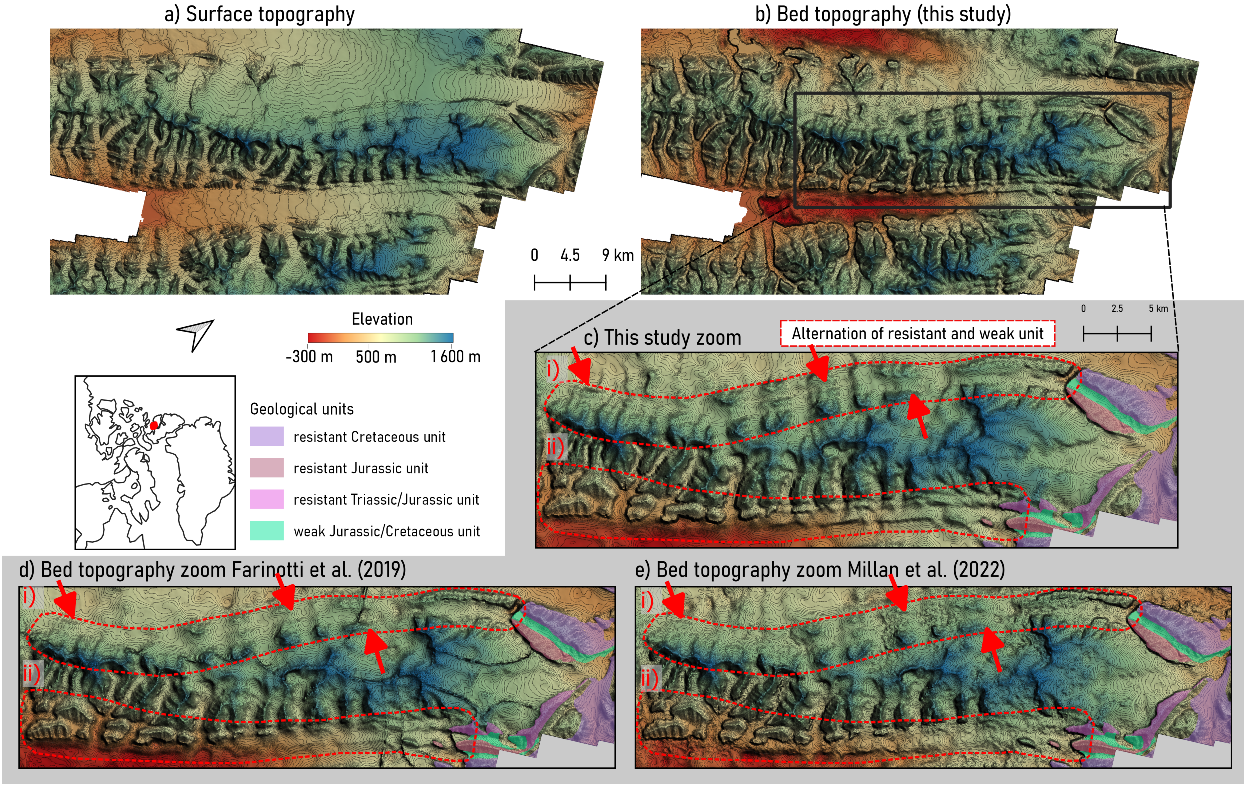

Figure S5: Example illustrating how simulated bed topography can aid geological mapping of currently glacierized terrain. a) Surface topography and b) simulated bed topography of Steacie ice cap, Axel Heiberg Island, Northern Canada. c) Close-up view of an area where an alternation of resistant and weak units mapped outside the ice cap [shown in pinkish and green colors, respectively; 8] is seen to continue under the present-day ice cover. This continuation is indicated by the red dashed outlines i) and ii). d and e) Bed topography from Farinotti et al. (2019) and Millan et al. (2022) of the same area. Whereas the longitudinal features in ii) can be seen in both products, too, as they are largely imprinted in ice-free topography, the continuation in i) is not visible in Millan et al. (2022) and barely visible in Farinotti et al. (2019). Red arrows point to example locations where the features seen in c) are much less pronounced or absent in d) and e).

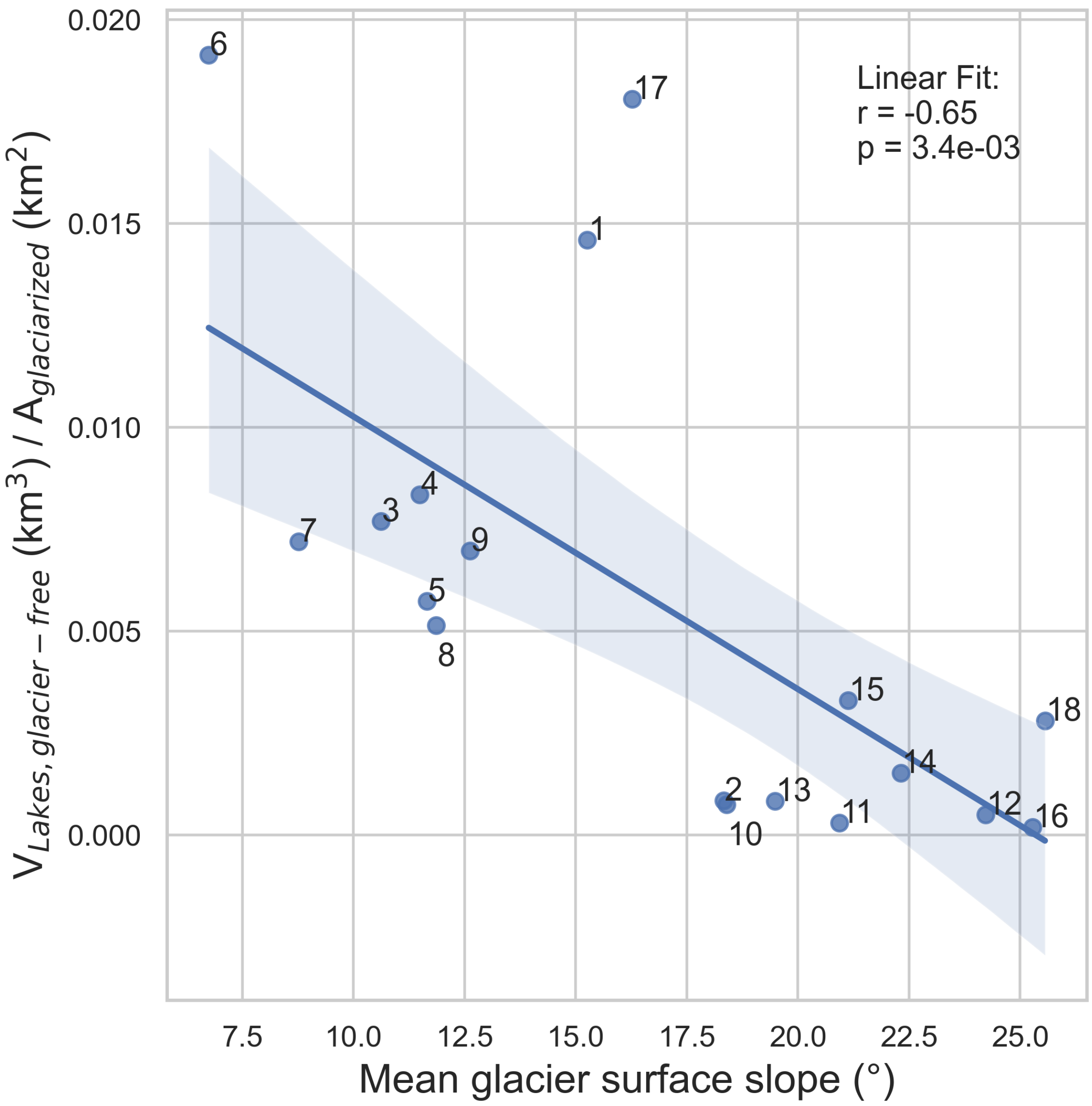

Figure S6: Potential glacier-free lake volume normalized by currently glacierized area over the mean glacier surface slope for each Randolph Glacier Inventory glacier region. Tidewater glaciers are not considered as subglacial overdeepenings connected to the ocean - frequently found at these glaciers - were not mapped as lakes. Linear fit indicates that regions with larger surface slopes have less potential for lakes. Annotated numbers denote Randolph Glacier Inventory glacier regions, and shading 95% confidence intervals.

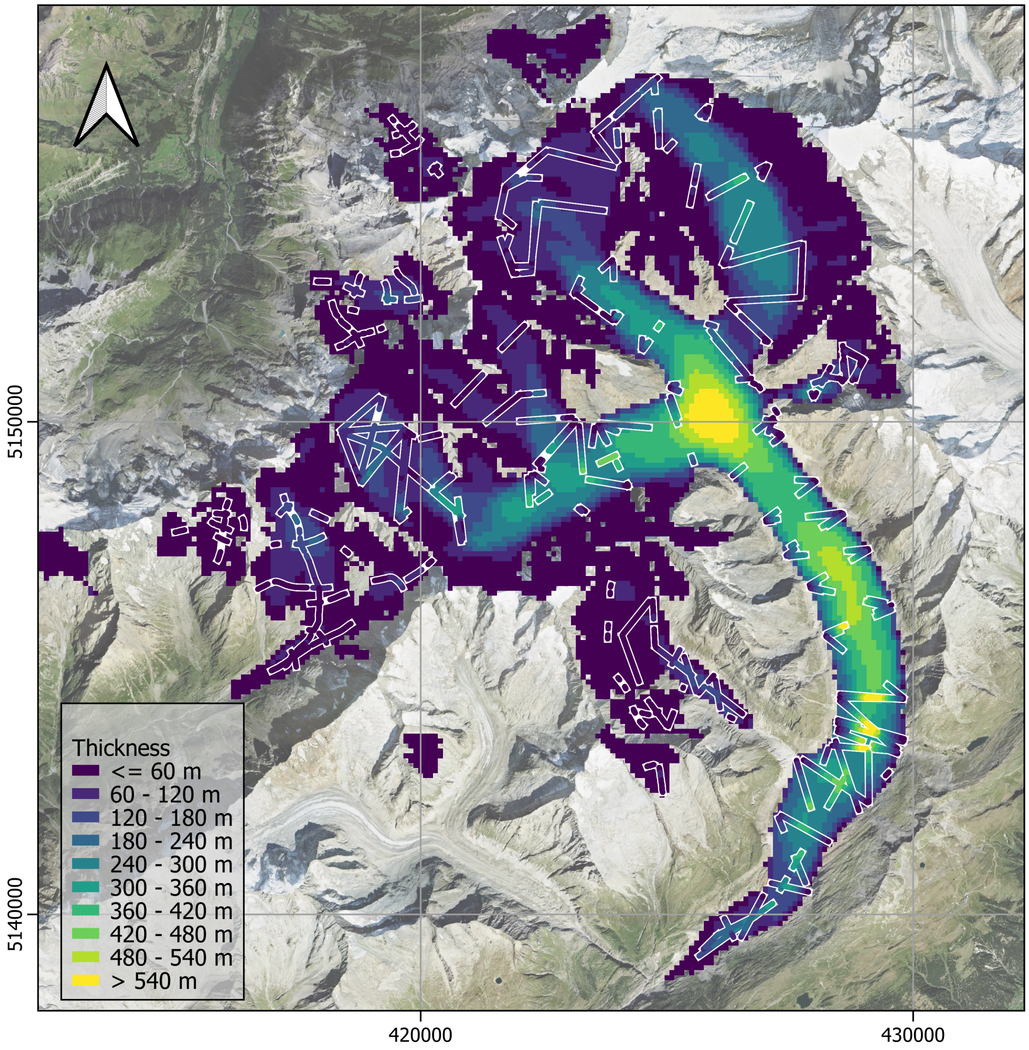

Figure S7: Modeled and observed ice thickness of Aletsch glacier and surrounding glaciers from the validation experiment (coordinate system: UTM 32N). All thickness observations on those glaciers (underlain by white lines) were not used to obtain these results. Background satellite imagery ©Google (2026).

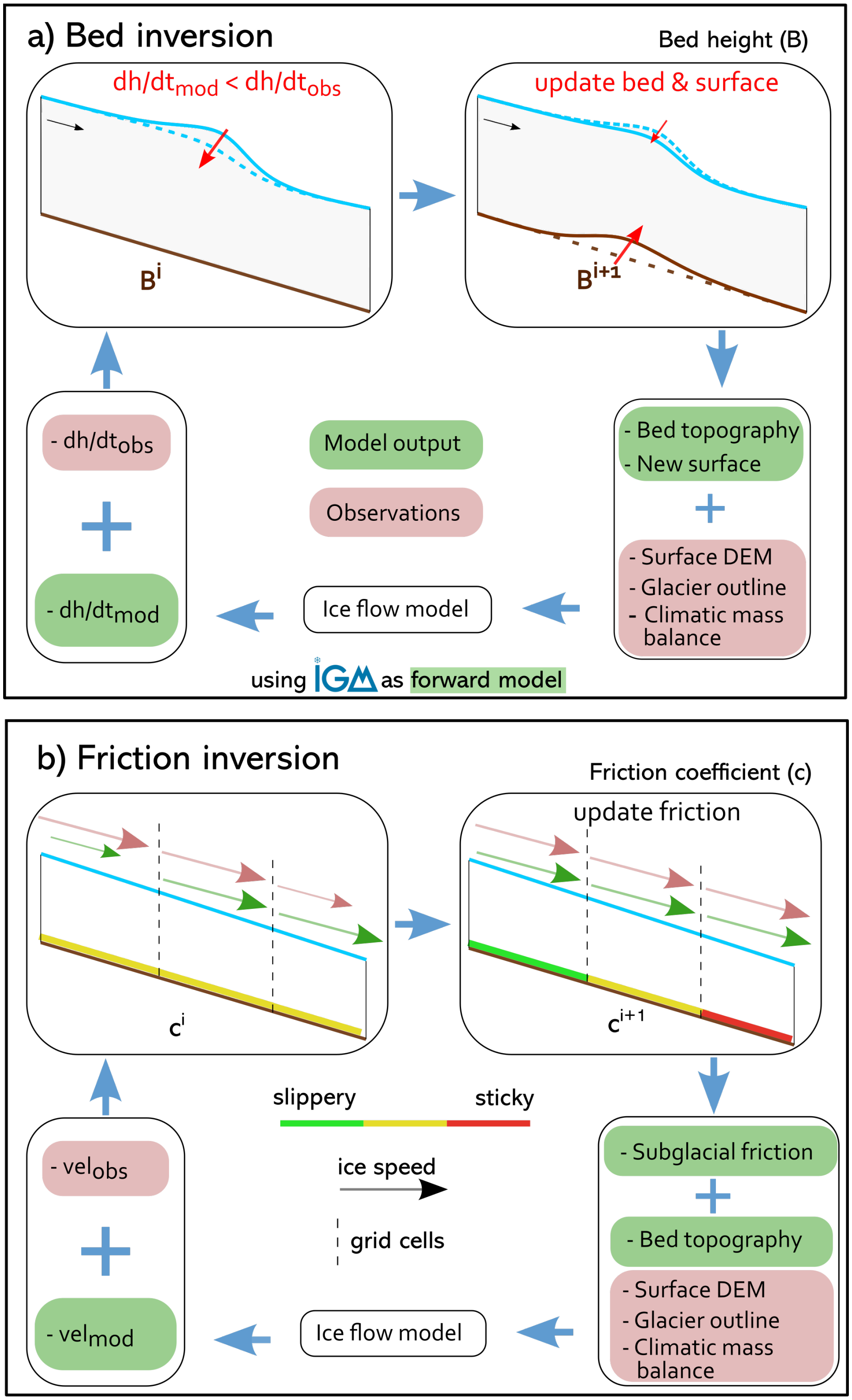

Figure S8: Illustration of the inversion methodology. a) Bed inversion where model output and observations are combined to iteratively update bed topography B, here illustrated for a glacier flowing from left to right with a surface shape indicated in blue. b) Friction inversion relying on velocity observations to infer the friction coefficient c.

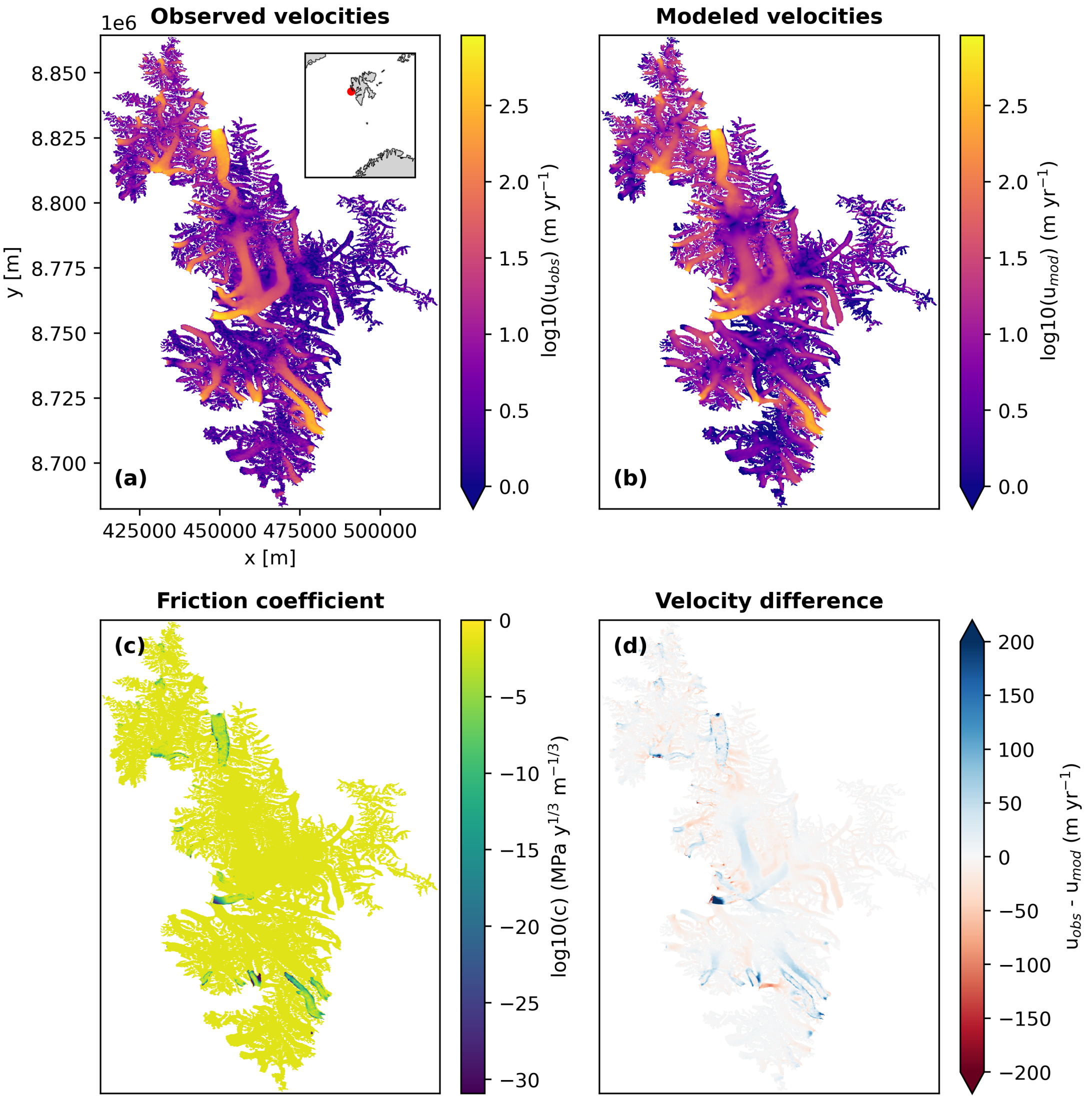

Figure S9: Observed and modeled velocities with inferred friction coefficient in a complex setting with multiple tidewater glaciers (coordinate system: UTM 33N). a) Observed [3] and b) modeled surface velocities in log scale with inset map showing location of glacier in Svalbard. c) Inferred friction coefficient in log scale. Values are uniform where the regionally tuned fixed value was applied, while values are variable where friction inversion with eq. (3) was done. d) Observed minus modeled velocities, showing generally low error where friction inversion was done.

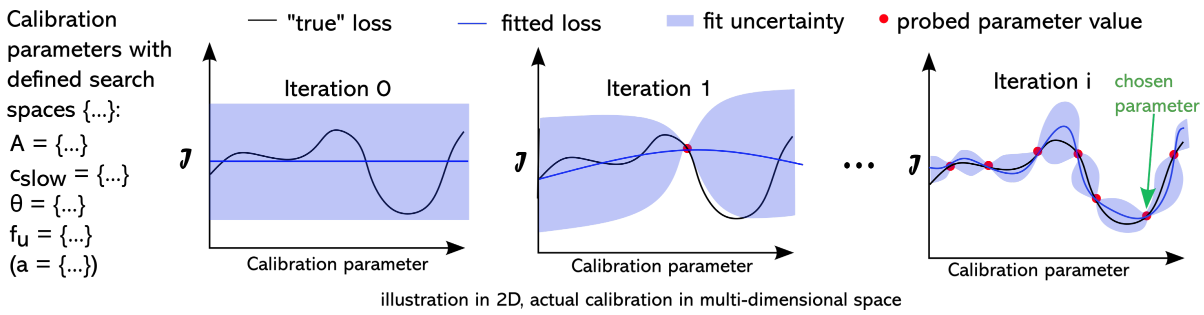

Figure S10: Illustration of calibration process. Over the course of several iterations i, the calibration parameters are varied within their predefined bounds to fit a loss function that minimizes the probability of having overlooked a minimum in the true loss function J. The chosen parameter combination is the one that yields the smallest loss.

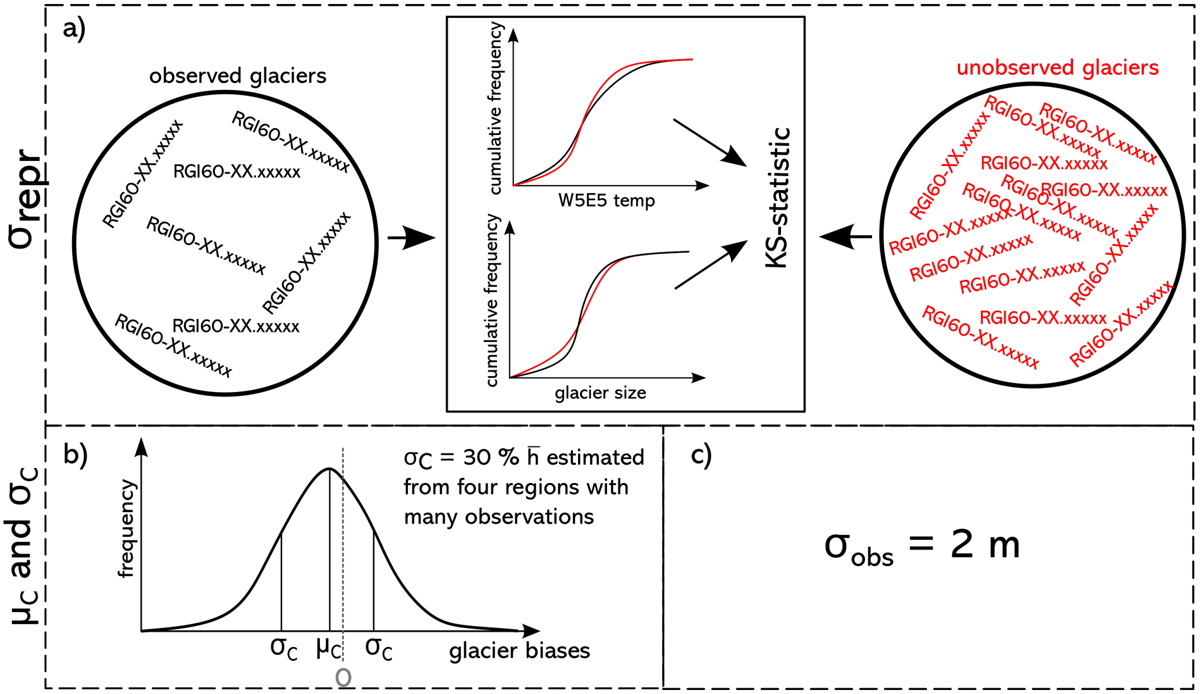

Figure S11: Illustration of the different error terms in eq. (6). a) The representativeness error σ_{repr} is based on comparing the cumulative density functions of observed and unobserved glaciers (here symbolized with Randolph Glacier Inventory glacier IDs) for two parameters, near-surface air temperature at the glacier from W5E5 [9, 10], and glacier size. The Kolmogorov-Smirnov (KS) test statistic is calculated in both cases as a measure of similarity. b) Derivation of σ_C and μ_C based on the histogram of glacier biases, where μ_C is their mean and σ_C their standard deviation. As described in the text, it is justified to set $\sigma_C = 30\%\bar{h}$ for all regions, based on extrapolation from regions Central Europe, Svalbard, Arctic Canada South and Greenland. c) The systematic bias of thickness observations is uniformly set to 2 m.

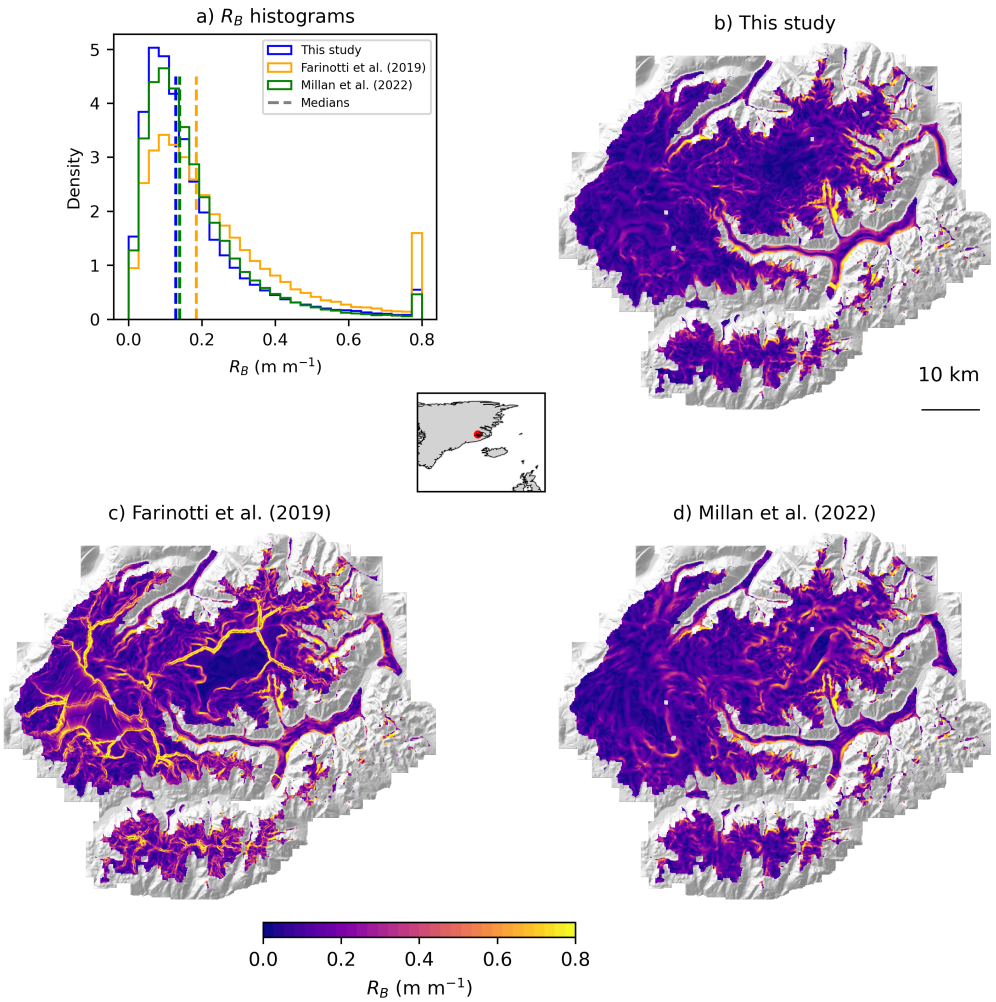

Figure S12: Comparison of bed roughness R_B with existing products for a glacier complex in Greenland (central coordinates of figure: 26.78°W 71.22°N). a) R_B histogram over the glaciarized area for this study (blue), [6] (yellow) and [3] (green), with dashed lines indicating median values. b) Map of R_B for this study. c) Map of R_B for [6] with large values above 0.8 m m^{-1} found at artificial walls between outlet glaciers, which are also reflected in the histogram. d) Map of R_B for [3].

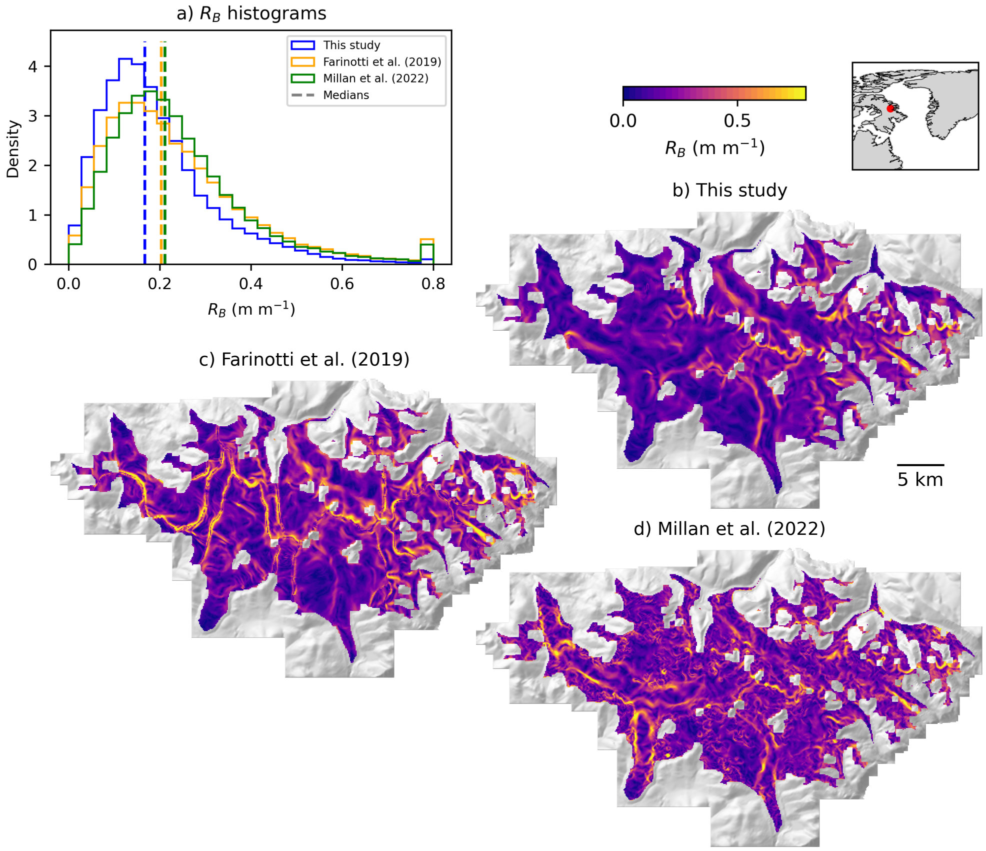

Figure S13: Comparison of bed roughness R_B with existing products for a glacier complex in Canada (central coordinates of figure: 69.70°W 69.39°N). a) R_B histogram over the glaciarized area for this study (blue), [6] (yellow) and [3] (green), with dashed lines indicating median values. b) Map of R_B for this study. c) Map of R_B for [6] with large values found at artificial walls between outlet glaciers, which are also reflected in the histogram. d) Map of R_B for [3] with high abundance of intermediate roughness values throughout the domain due to an overall noisy bed shape.

Supplementary Methods

Inversion method

Figure S8 schematically illustrates the inversion process. The Instructed Glacier Model [IGM; 11] is initialized with distributed fields of surface elevation, climatic mass balance and bed topography (Fig. S8a). IGM models the ice flow dynamics using a Convolutional Neural Network (CNN), trained to satisfy higher-order (Blatter-Pattyn) ice flow equations [12]. At each inversion iteration, IGM updates the weights of the CNN to adapt to new conditions (bedrock and sliding coefficient), ensuring consistency with higher-order ice flow physics [11]. The initial guess of bed topography is taken from [7], but smoothed heavily. Previous work has shown that the influence of initial conditions on the final solution scales with the regularization parameter θ (eq. (2), [13, 14]); $\theta = 0$ yields fully independent solutions. Here, θ is calibrated to achieve maximum inversion performance. With $\theta = 0.3$, the highest value used here (Table S3), the resulting bed geometry may retain some features of the initial guess, though this has only a small effect on the calibrated ice volume, which is overwhelmingly constrained by thickness observations. Additional model parameters include the ice viscosity η - a calibration parameter - and a friction coefficient, also calibrated as one spatially uniform value for slow-flowing areas of marine-terminating glaciers and all land-terminating glaciers, but inverted in fast-flowing areas of marine-terminating glaciers (see below). The latter are here defined as glaciers with ice-covered grid cells adjacent to a grid cell with surface elevation < 5 m a.s.l..

The initialized model is run forward over a short time step dt, the maximum duration of which is determined by IGM based on the Courant-Friedrichs-Lewy condition (Fig. S8a). Modeled and observed dh/dt are compared, and the bed is updated according to eq. (1). For example, in the case shown in Fig. S8a, $dh/dt_{mod} < dh/dt_{obs}$, resulting in a bed uplift. If $\theta > 0$, the regularization term (Eq.(2)) simultaneously applies a corresponding surface lowering at the same location. This has previously been shown to evenly distribute the ice and avoid small-scale bed irregularities not justified by the input data [13]. This cycle of forward modeling, comparison, and bed/surface adjustment is repeated over several thousand iterations to reach convergence.

Friction updates are required much less frequently [13], but critically help to align model and observed dynamics in fast-flowing areas of marine-terminating glaciers (Fig. S8b). Applying the same principle as above, in this case, modeled and observed velocities are compared instead of dh/dt, and the friction coefficient c is updated instead of bed topography (eq. (3)). In grid cells where $u_{obs} > u_{mod}$, the bed is made more slippery, whereas in the opposite case, the bed is made more sticky (Fig. S8b). No explicit regularization is applied to the friction field. An example from Svalbard (Fig. S9) shows observed and modeled velocities alongside the inverted friction coefficient. The velocity difference is generally small where the friction coefficient was inverted.

Input data preparation

Gridded inputs of dh/dt [15] representing the period 2000-2019, surface topography from the Copernicus 90 m DEM [acquired between 2011 and 2015; 16], velocity observations (either [3] or ITS_LIVE [17], with time stamps between 1985 and 2018) and the initial bed [7] are obtained for each glacier using the OGGM shop [18]. Glacier outlines are from the RGI v6 [mean date: 2002; 1, 2], here used in a slightly updated version that comes with OGGM which corrects obvious errors. The dh/dt observations are converted to mass changes assuming a density of 850 kg m^{-3} [19] to make them comparable to IGM output. Modeled climatic mass balance gradients also representing 2000-2019 are taken from [20] and projected onto the glacier grid by applying them to the surface elevations of the DEM. To close the mass budget of a glacier inside its domain Ω, it is required that

$$\int_\Omega \dot{b(z)} - \int_\Omega \frac{dh}{dt}(z) + e = a, \tag{S1}$$

where $a > 0$ is frontal ablation of marine-terminating glaciers, $\dot{b}$ is specific climatic mass balance, z is surface elevation and e is an error term. The latter reflects that relying on the different observational (and modeled, in the case of $\dot{b}$) datasets alone often would not satisfy the equation with $e = 0$. Therefore, we calculate e for each glacier, thus ensuring mass budget closure. As in [5], we fit a piecewise linear function over z to Eq. (S1) to obtain fields of $\dot{b}$ and dh/dt that increase monotonically with elevation and are consistent with each other.

Surface topography in the ablation area is smoothed before the inversion using a Gaussian filter. Debris-covered areas as identified by [21] receive additional smoothing to mitigate spurious effects resulting from high topographic variability induced by surface processes.

In RGI region 5 (Greenland Periphery), only glaciers with connectivity level 0 and 1 [2] are considered as in previous global glacier studies [4]. For glaciers for which no masks are provided in the OGGM shop (i.e. very small ones or without proper polygon in the RGI) we also do not produce any outputs.

Calibration

Fig. S10 shows a schematic of the Bayesian calibration where the cost function J (eq. (4)) is minimized by varying the calibration parameters within predefined bounds. Probing different parameter values over several iterations and calculating a fitted loss function with uncertainties (no uncertainty at probed parameter values), the algorithm aims to minimize the likelihood of having overlooked the minimum in the "true" loss. Nonetheless, there is no guarantee of finding it. At least 30 calibration iterations are performed per RGI region. A comparison to independent manual calibration for four RGI regions showed that this suffices to find parameters that are as good or better than manual tuning. To reduce computational cost, calibration is carried out at half the resolution of production runs.

The thickness observations from the GlaThiDa used during calibration are corrected for ice thickness

changes since data acquisition by calculating the difference between the GlaThiDa surface elevation and the input DEM. In High Mountain Asia (RGI regions 13-15), this is not done because cross-validation between original and recomputed ice thicknesses showed unrealistic values for the latter, likely due to geolocation errors of the observations or errors in the DEM. Where no information on surface elevation is stored in the GlaThiDa [$\sim$ 12% of observations; 22], this is also not possible. The mean acquisition date of all raw GlaThiDa point observations is autumn 2011 (median: spring 2013); after corrections, this is approximately 2013, given that the DEM is from 2011-2015. This time stamp is roughly consistent with the mean year of the dh/dt and mass balance inputs (2010), but mismatched by around a decade with the glacier outlines (2002). Since we tie the modeled volumes to the thickness observations through the calibration process, we refer to our computed volumes as approximately representing the year 2013. However, due to the above inconsistencies which are unavoidable with current input data availability, individual glacier thicknesses may represent a larger time span (approximately 2000 - 2015).

RGI regions Iceland, Russian Arctic and South Asia West do not have any point observations of ice thickness in the GlaThiDa. Therefore, for Iceland, we take the mean of the Greenland and Svalbard model parameters. For the Russian Arctic, we choose the ones from Svalbard, and for South Asia West, we apply the parameters from Central Asia. For Iceland and the Russian Arctic, we validate and find good performance against observations of mean thickness that are available for some glaciers in these regions in the GlaThiDa (Fig. S1). For South Asia West, since no mean thicknesses are reported, we cannot validate our results.

In RGI region Alaska, we find that our calibration method produces parameters that lead to unrealistically thin ice for most glaciers, although the thickness in the thick parts of the large ice fields bordering the Gulf of Alaska, where the majority of thickness observations are located, is overestimated. We attribute this to a combination of surging and extremely fast glaciers (e.g. Columbia glacier) included in the calibration glaciers, and to low surface slopes in the interior of the ice fields, all of which make the calibration more uncertain. To represent the bulk of the glaciers correctly, we manually select parameters in this region such that thin to medium-thick glaciers are represented more accurately, at the cost of overestimating the ice thickness in the thick areas of the ice fields (Fig. S1). Accordingly, the volume uncertainty for Alaska is high, although we bound μ_C (eq. (6)) at -100 m to not produce unreasonably large errors.

Post-processing

Small areas within the glacier outlines remaining ice-free after the inversion are particularly found towards the glacier front. This is because mass leakages through the glacier boundaries mean that the glacier is "running out of ice" at the front. To minimize this effect we buffer the glacier masks by two grid cells during the inversion, allowing ice to exit and reenter the glacier intermittently. Ice-free areas on land-terminating glaciers are interpolated using a perfect-plasticity approach [23] where the yield stress is determined as the median of the ice-covered grid cells multiplied by 0.8 to account for often weaker sub-glacial sediments in the lower reaches of glaciers. Ice-free areas of marine-terminating glaciers are filled with bilinear interpolation.

For all glaciers, as a final step after the inversion, the modeled thicknesses are smoothed with a thickness-dependent Gaussian filter to preserve detail in thin areas and smooth unjustified bed roughness in thick areas as in [5]. Final bed elevations B_{mod} are then produced by subtracting the thicknesses h_{mod} from the final glacier surface in the model $S^{i_{max}}$. Note that, due to eq. (2), $B_{mod} + h_{mod} = S^{i_{max}} \neq S_{obs}$, implying that modeled bed elevations plus modeled thicknesses do not add up to the observed surface elevations. We favor this inequality over adjusting B_{mod} or h_{mod} artificially because 1) it yields an output consistent with the model setup, and 2) DEMs are known to contain significant errors in mountainous terrain [24].

Uncertainty estimation

We here provide additional information on the different terms in eq.(6).

To account for non-representativness of the glaciers with observations compared to all glaciers in a given RGI region, we devise a relationship

$$\sigma_{repr} = \bar{h}\,(D_{temp} \cdot \lambda_{temp} + D_{area} \cdot \lambda_{area}) \quad \text{(S2)}$$

where D denotes the test statistic of the non-parametric, two-sample Kolmogorov-Smirnov (KS) test, a common measure for similarity between samples with a range between 0 (two identical samples) and 1 (completely different), calculated based on the cumulative probability functions of the two samples (Fig. S11a). The two samples here are observed and unobserved glaciers. D_{temp} is the KS statistic using the mean air temperature at the glaciers as a proxy for climatological differences, taken from W5E5 [9, 10] over the period 2000-2019 and adjusted for the elevation difference between median glacier elevation and W5E5 pixel elevation assuming a lapse rate of 6 K km^{-1}. D_{area} is the equivalent using the glacier area as a proxy for ice-dynamical differences. The weights $\lambda_{temp} = 0.2$ and $\lambda_{area} = 0.05$ are here chosen to reflect that we consider area differences to be less problematic than climatological differences. This is because larger glaciers tend to be better observed than small ones, which in fact is advantageous given that they are generally more voluminous [25], and hence more important for constraining total volume. Eq. (S2) is entirely empirical and only serves to yield a first-order estimate of how representative the observed glaciers are of all glaciers in an RGI region. As an example, $\sigma_{repr} = 5.7$ m (12% $\bar{h}$) for RGI region North Asia with only $n_{obs} = 4$ out of $n_{total} = 5151$ glaciers being observed and $\bar{h} = 49$ m, while on Svalbard with many spatially distributed thickness observations concentrated on large glaciers ($n_{obs} = 207$, $n_{total} = 1615$, $\bar{h} = 202$ m) $\sigma_{repr} = 10.3$ m (5% $\bar{h}$).

The sampling uncertainty $\frac{\sigma_C}{\sqrt{n_{obs}}}$ and the calibration bias μ_C both derive from the histogram of glacier biases (Fig. S11b). Glacier biases here refers to, for each glacier, the mean difference between observed and modeled thicknesses at the locations of thickness observations. The mean of these biases is μ_C and their standard deviation is σ_C. The latter can be estimated well for RGI regions with many observed glaciers, but not for small sample sizes. To alleviate this issue, we calculate σ_C in four RGI regions with many observed

glaciers, considering only glaciers with at least ten thickness observations, and find that it scales with mean thickness $\bar{h}$ (Central Europe: $\sigma_C = 27\% \ \bar{h}$, Svalbard: $\sigma_C = 30\% \ \bar{h}$, Arctic Canada South: $\sigma_C = 20\% \ \bar{h}$ and Greenland: $\sigma_C = 26\% \ \bar{h}$). Through least-squares fitting, we establish that $\sigma_C <= 30\% \ \bar{h}$ (95% probability) which is hence the value we use also for regions with few observations.

The systematic observation error σ_{obs} can generally be assumed to be small with the main error source being systematic errors in the time-to-depth conversion of radar measurements. For simplicity, we set it uniformly to $\sigma_{obs} = 2$ m (Fig. S11c), knowing that regions observed by many different research groups are less likely to have systematic errors compared to ones where all observations come from a small group of researches that may make similar assumptions, e.g. about wave propagation velocity. Note that the definition of *systematic* errors is different from point thickness uncertainty of radar observations which are often on the order of a few tens of meters.

Supplementary Discussion

Quantitative comparison between thickness products

To quantitatively compare the thickness products from this study, [7] and [3] we calculate their agreement with GlaThiDa observations [22, 26] at the global scale. We obtain MAEs of 122 m (ME: 33 m) for [7], 106 m (ME: 11 m) for [3], and 94 m (ME: 12 m) for this study (Fig. S2), considering for each study only those glaciers for which it produced an output.

However, these numbers need to be interpreted with care due to the lack of a shared pool of independent validation data. A definitive assessment of model performance requires observations that were not used for model calibration. In practice, all studies used the full set of available GlaThiDa observations for that purpose. Moreover, only few thickness measurements have become available in recent years that are not yet included in GlaThiDa [26], and these new data remain too sparse to support a comprehensive global evaluation. This precludes a fully independent quantitative assessment of model performance.

Meaningful intercomparison in the absence of independent validation data depends on whether the degree to which different calibration approaches allow models to generalize from observed to unobserved locations is comparable. This generalization skill is closely related to the effective number of degrees of freedom in the calibration. Approaches based on the adjustment of a limited set of region-wide parameters impose strong structural constraints and therefore favor generalization, whereas methods that locally assimilate individual measurements introduce substantially more degrees of freedom, enabling very close agreement with observations but increasing the risk of overfitting.

Under the assumption that different studies generalized the information from observed to unobserved locations in a broadly comparable manner, a ranking based on performance at observed locations can be informative. This study and the work by [7] and [3] all relied predominantly on region-wide parameter calibration with only a few exceptions, such as one model in the ensemble approach by [7] that locally assimilated thickness

observations [27]. Given the otherwise similar calibration strategies we find the above metrics indicative of model performance, though not fully conclusive in the absence of independent validation data.

Identifying artifacts in existing products on a large scale

In Figs. S3, S4, we show examples of unrealistic bed topography found in the products by [7] and [3]. These manifest predominantly as ”walls” between outlet glaciers of glacier complexes and highly irregular bed topography on small glaciers. To quantitatively describe these artifacts, we compute the bed roughness as the root squared slope

$$R_B = \sqrt{\left(\frac{dB}{dx}\right)^2 + \left(\frac{dB}{dy}\right)^2} \tag{S3}$$

for the shown examples (Figs. S12, S13) and all glaciers globally, for this study and existing products [3, 7]. In Figs. S12c and S13c, it is evident that the ”walls” between outlet glaciers show up as very large R_B values, due to the sudden jumps in bed height. These many large values result in a spike in [7]´s R_B histogram for $R_B \geq 0.8$ m m^{-1} (Figs. S12a and S13a). In Fig. S13d, we find that the product by [3] exhibits elevated R_B values across most of the domain where this study and [7] show a smooth bed. This can be traced back to the overall noisy bed appearance seen in Fig. S4d. Going beyond these two examples to the global scale, we find that the product by [7] has the largest mean bed roughness for large glaciers (>10 km^2) whereas [3] is the roughest for small (<=10 km^2) glaciers. We interpret this to reflect the patterns seen in the examples discussed here (Figs. S12 and S13): For large glacier complexes with many outlet glaciers, the artificial ”walls” produced by [7] make the bed unrealistically rough. Meanwhile, for small glaciers, the small-scale bed noise introduced by large relative uncertainties in mapping slow ice flow velocities results in too-rough beds in [3]. This quantitative analysis demonstrates that the artifacts seen in Figs. S3, S4, which TOPO-DE is free of, impact the realism of the existing bed products on a large scale. Note that the elevated roughness of the products by [7] and [3] described here does not contradict possibly too smooth bed shapes indicated by the thickness-dependent biases seen in Fig. 2: the roughness quantifies bed variations from one grid point to the next whereas the previously described biases concern patterns on a larger scale, such as the systematic underestimation of the depth of subglacial troughs and the height of bed peaks.

Uncertainties and Limitations

Biases in the dh/dt and mass balance products, most likely to be found in regions with few mass balance observations [28], lead to volume biases of individual glaciers. Errors in the conversion from elevation to mass change act in the same manner. However, the non-linear ice flow physics render the influence of such mass flux errors on modeled thicknesses and volumes small [13]. Erroneous glacier outlines that encompass ice-free terrain introduce a geometrical inconsistency between modeling domain and actual ice cover, typically resulting in overestimated ice thickness. Ice-free terrain as seen in a DEM may also show up inside glacier outlines if a DEM has a later acquisition date than the outlines, due to glacier retreat. On glacier complexes,

incorrect ice divides compromise the mass budget closure of individual outlet glaciers because the delineated catchments do not correspond to the actual flow directions. This leads to excess mass in one outlet and mass deficits in another, with corresponding thickness biases. Mass shortages result in zero ice thicknesses near the glacier front - such areas are filled using a perfect-plasticity approach (see above) or by interpolation with associated elevated uncertainties. In fast-flowing areas of marine-terminating glaciers where we invert for basal friction ice velocity errors lead to too thick and too thin ice for negative and positive biases, respectively [13]. Bed shape errors are introduced where observed surface elevations correspond to features not produced by ice dynamics but by surface processes (e.g. large snow drifts, medial moraines, and debris). Our input preparation workflow (Supplementary Methods) is designed to minimize the influence of input data errors as much as possible though residual artifacts naturally remain. Thanks to higher-order ice flow physics, TOPO-DE suffers much less from shortcomings due to unmodeled processes than previous SIA-based studies that ignored horizontal stress gradients [3, 7]. However, we expect elevated errors for surging glaciers for which a comprehensive flow law is missing [29]. Furthermore, different time stamps of input datasets are problematic for surging glaciers because fast dynamical changes require highly time-synchronized inputs for accurately obtaining bed elevations [14]. The unavailability of a global inventory of lake-terminating glaciers forces us to model such glaciers as land-terminating, contributing to increased errors at and near their fronts. Finally, ice thickness inversions are generally underconstrained [30], implying that several solutions may explain the observations. Bed features smaller than at least one ice thickness cannot be resolved, setting a physical limit to the bed detail of any inversion [31, 32]. The modeled lakes are potentially influenced by all these challenges, introducing unavoidable uncertainty on their actual location and volume.

Supplementary References

1. Pfeffer, W. T. *et al.* The Randolph Glacier Inventory: a globally complete inventory of glaciers. *Journal of Glaciology* **60,** 537–552. doi:10.3189/2014JoG13J176 (2014).
2. RGI Consortium. *Randolph Glacier Inventory – A Dataset of Global Glacier Outlines: Version 6.0* (NSIDC: National Snow and Ice Data Center, Boulder, Colorado USA, 2017).
3. Millan, R., Mouginot, J., Rabatel, A. & Morlighem, M. Ice velocity and thickness of the world's glaciers. *Nature Geoscience* **15,** 124–129. doi:10.1038/s41561-021-00885-z (Feb. 2022).
4. Hock, R., Maussion, F., Marzeion, B. & Nowicki, S. What is the global glacier ice volume outside the ice sheets? *Journal of Glaciology* **69,** 204–210. doi:10.1017/jog.2023.1 (Feb. 2023).
5. Frank, T. & van Pelt, W. J. J. Ice volume and thickness of all Scandinavian glaciers and ice caps. *Journal of Glaciology,* 1–14. doi:10.1017/jog.2024.25 (Mar. 2024).
6. Farinotti, D., Round, V., Huss, M., Compagno, L. & Zekollari, H. Large hydropower and water-storage potential in future glacier-free basins. *Nature* **575,** 341–344. doi:10.1038/s41586-019-1740-z (Nov. 2019).

7. Farinotti, D. *et al.* A consensus estimate for the ice thickness distribution of all glaciers on Earth. *Nature Geoscience* **12,** 168–173. doi:10.1038/s41561-019-0300-3 (Mar. 2019).

8. Harrison, J. C. & Jackson, M. P. *Bedrock geology, Strand Fiord-Expedition Fiord area, western Axel Heiberg Island, northern Nunavut (parts of NTS 59E, F, G, and H)* tech. rep. 5590 (Geological Survey of Canada, 2008).

9. Cucchi, M. *et al.* WFDE5: bias-adjusted ERA5 reanalysis data for impact studies. *Earth System Science Data* **12,** 2097–2120. doi:10.5194/essd-12-2097-2020 (Sept. 2020).

10. Lange, S. *et al. WFDE5 over land merged with ERA5 over the ocean (W5E5 v2.0)* 2021. doi:10.48364/ISIMIP.342217.

11. Jouvet, G. & Cordonnier, G. Ice-flow model emulator based on physics-informed deep learning. *Journal of Glaciology,* 1–15. doi:10.1017/jog.2023.73 (Sept. 2023).

12. Blatter, H. Velocity and stress fields in grounded glaciers: a simple algorithm for including deviatoric stress gradients. *Journal of Glaciology* **41,** 333–344. doi:10.3189/S002214300001621X (1995).

13. Frank, T., van Pelt, W. J. J. & Kohler, J. Reconciling ice dynamics and bed topography with a versatile and fast ice thickness inversion. *The Cryosphere* **17,** 4021–4045. doi:10.5194/tc-17-4021-2023 (Sept. 2023).

14. Van Pelt, W. & Frank, T. New glacier thickness and bed topography maps for Svalbard. *The Cryosphere* **19,** 1–17. doi:10.5194/tc-19-1-2025 (Jan. 2025).

15. Hugonnet, R. *et al.* Accelerated global glacier mass loss in the early twenty-first century. *Nature* **592,** 726–731. doi:10.1038/s41586-021-03436-z (Apr. 2021).

16. ESA & Airbus. *Copernicus DEM – Global and European Digital Elevation Model (COP-DEM)* 2019. doi:https://doi.org/10.5270/ESA-c5d3d65.

17. Gardner, A., Fahnestock, M. & Scambos, T. *MEaSUREs ITS_LIVE Landsat Image-Pair Glacier and Ice Sheet Surface Velocities, Version 1* Boulder, Colorado USA, 2022. doi:10.5067/IMR9D3PEI28U.

18. Maussion, F. *et al.* The Open Global Glacier Model (OGGM) v1.1. *Geoscientific Model Development* **12,** 909–931. doi:10.5194/gmd-12-909-2019 (Mar. 2019).

19. Huss, M. Density assumptions for converting geodetic glacier volume change to mass change. *The Cryosphere* **7,** 877–887 (2013).

20. Rounce, D. R. *et al.* Global glacier change in the 21st century: Every increase in temperature matters. *Science* **379,** 78–83. doi:10.1126/science.abo1324 (Jan. 2023).

21. Scherler, D., Wulf, H. & Gorelick, N. Global Assessment of Supraglacial Debris-Cover Extents. *Geophysical Research Letters* **45,** 11, 798–11, 805. doi:10.1029/2018GL080158 (2018).

22. Welty, E. *et al.* Worldwide version-controlled database of glacier thickness observations. *Earth System Science Data* **12,** 3039–3055. doi:10.5194/essd-12-3039-2020 (Nov. 2020).

23. Nye, J. F. The flow of glaciers and ice-sheets as a problem in plasticity. *Proceedings of the Royal Society of London. Series A. Mathematical and Physical Sciences* **207,** 554–572 (1951).

24. Chen, W. *et al.* Towards ice-thickness inversion: an evaluation of global digital elevation models (DEMs) in the glacierized Tibetan Plateau. *The Cryosphere* **16,** 197–218. doi:10.5194/tc-16-197-2022 (Jan. 2022).

25. Bahr, D. B., Meier, M. F. & Peckham, S. D. The physical basis of glacier volume-area scaling. *Journal of Geophysical Research: Solid Earth* **102,** 20355–20362. doi:10.1029/97JB01696 (1997).

26. GlaThiDa Consortium. *Glacier Thickness Database 3.1.0* Zürich, Switzerland, 2020.

27. Fürst, J. J. *et al.* Application of a two-step approach for mapping ice thickness to various glacier types on Svalbard. *The Cryosphere* **11,** 2003–2032. doi:10.5194/tc-11-2003-2017 (Sept. 2017).

28. WGMS. *Fluctuations of Glaciers Database* World Glacier Monitoring Service, Zürich, Switzerland, 2022. doi:10.5904/wgms-fog-2022-09.

29. Thøgersen, K., Gilbert, A., Schuler, T. V. & Malthe-Sørenssen, A. Rate-and-state friction explains glacier surge propagation. *Nature Communications* **10,** 2823. doi:10.1038/s41467-019-10506-4 (June 2019).

30. Bahr, D. B., Pfeffer, W. T. & Kaser, G. Glacier volume estimation as an ill-posed inversion. *Journal of Glaciology* **60,** 922–934. doi:10.3189/2014JoG14J062 (2014).

31. Raymond, M. J. & Gudmundsson, G. H. On the relationship between surface and basal properties on glaciers, ice sheets, and ice streams. *Journal of Geophysical Research: Solid Earth* **110.** doi:10.1029/2005JB003681 (2005).

32. Gudmundsson, G. H. & Raymond, M. On the limit to resolution and information on basal properties obtainable from surface data on ice streams. *The Cryosphere* **2,** 167–178. doi:10.5194/tc-2-167-2008 (Dec. 2008).
